# Needling With Continuous Infusion Into the Anterior Chamber in Eyes with Bleb Failure After PreserFlo MicroShunt Implantation

**DOI:** 10.7759/cureus.94188

**Published:** 2025-10-09

**Authors:** Katsuhiko Maruyama

**Affiliations:** 1 Ophthalmology, Yashio Maruyama Eye Clinic, Saitama, JPN

**Keywords:** anterior chamber, anterior chamber infusion, anterior chamber maintainer, bleb needling, continuous infusion, filtration failure, infusion, microshunt, needling, preserflo microshunt

## Abstract

The PreserFlo MicroShunt (PMS) is a minimally invasive glaucoma device; however, filtration failure due to subconjunctival fibrosis remains a clinical challenge, and the role of bleb needling after PMS implantation remains unclear. This study evaluated the safety and efficacy of bleb needling combined with continuous infusion into the anterior chamber to treat filtration failure after PMS implantation. Four consecutive pseudophakic eyes underwent the procedure, which involved anterior chamber infusion with balanced salt solution and lysing adhesions with a 27-gauge needle under surgical microscopy. No intraoperative complications were observed. During follow-up, repeat needling was required in three eyes, and only one eye required the addition of intraocular pressure (IOP) lowering medication. None of the eyes underwent additional glaucoma surgery, and IOP was lower than that before the PMS implantation. No ocular infections occurred throughout the clinical course. These findings suggest that, although the IOP-lowering effect of needling with continuous infusion into the anterior chamber after PMS implantation is limited compared to trabeculectomy, the technique may help maintain IOP control and avoid reoperation in select cases. The method also allows for safer manipulation by estimating tube position and performing adhesion lysis under controlled irrigation. However, its use is restricted by the need for operating room facilities, resource demands, and limited insurance coverage. While this small case series cannot establish definitive conclusions, the results suggest that bleb needling with anterior chamber infusion may represent a useful alternative to reoperation for PMS filtration failure, which warrants further investigation in larger studies.

## Introduction

The PreserFlo MicroShunt (PMS) (Santen Pharmaceutical, Co., Ltd., Osaka, Japan) is an 8.5-mm-long glaucoma surgical device with a lumen diameter of 70 μm. It is made from a highly biocompatible, bioinert material called poly (styrene-block-isobutylene-block-styrene) or SIBS. The PMS is used in filtration surgery to reduce intraocular pressure (IOP) by draining aqueous humor from the anterior chamber into the subconjunctival space [1]. Although PMS implantation is less effective in lowering IOP than trabeculectomy [2,3], it may benefit patients due to a lower risk of hypotony-related events [2] or a lower reintervention rate [3]. Because PMS implantation does not require the creation of a scleral flap, ostomy, peripheral iridectomy, and the suturing of a scleral flap, as is necessary for trabeculectomy, it is also a less invasive and more standardized procedure [4]. Consequently, PMS implantation is increasingly performed as an alternative to trabeculectomy in Japan [5].

Since PMS implantation involves a filtration surgery, a certain percentage of eyes are destined to develop filtration failure due to fibrosis of scar tissue after surgery. Several methods can be used to manage increased IOP postoperatively, including steroids, non-steroidal anti-inflammatory drugs, IOP-lowering medications, bleb needling, and open revision [6,7]. Of these methods, needling is a procedure that can be expected to produce immediate effects by directly addressing the cause of filtration failure, and despite being a surgical technique, it is minimally invasive. However, needling after PMS implantation is challenging, and few physicians believe that sufficient IOP reduction can be achieved with needling alone. PMS implantation experts also agree that revision surgery is preferable to bleb needling when filtration failure occurs postoperatively [6,7]. On the other hand, both the surgeon and the patient must give significant consideration to the decision to undergo open bleb revision and the wish to avoid it if possible. In fact, a meta-analysis found that no statistical difference was observed in the number of patients requiring needling after PMS implantation or trabeculectomy [2]. Many physicians believe that if there is an effective needling method that can facilitate adhesion lysis and visualization of outflow, it is worth trying.

The bleb needling procedure, augmented with continuous infusion into the anterior chamber, has been reported for eyes with filtration failure after trabeculectomy [8,9]. Applying a similar method to eyes with filtration failure after PMS implantation may solve the problem. The aim of this report is to evaluate the efficacy and safety of the procedure used to treat filtration failure after PMS implantation.

## Technical report

Subjects

This technical report included four consecutive eyes (four cases) that developed filtration failure after PMS implantation and underwent bleb needling with continuous infusion into the anterior chamber. This study was approved by the Ethics Review Committee of the Japan Medical Association (No. R5-14). Table 1 shows the patient characteristics at baseline.

**Table 1:** Patient characteristics at baseline *Fix combination eye drops or oral carbonic anhydrase inhibitors count as two medications. **The duration between open bleb revision following PreserFlo MicroShunt implantation and needling was 10 months. IOP, intraocular pressure; PMS, PreserFlo MicroShunt; BCVA, best-corrected visual acuity; ECD, endothelial cell density; SOAG, secondary open-angle glaucoma; PACG, primary angle-closure glaucoma; POAG, primary open-angle glaucoma

No.	Age (years)	Glaucoma type	IOP before PMS implantation (mmHg)	Number of medications^*^	Duration between PMS implantation and needling (month)	IOP at the time of needling (mmHg)	BCVA (logMAR)	ECD (cell/mm^2^)
1	80	SOAG	20	5	9	17	0	2,400
2	84	PACG	20	2	1	22	0	1,431
3	55	POAG	38	2	12^**^	16	-0.1	2,522
4	74	SOAG	19	3	4	18	-0.1	2,489

All of the eyes were pseudophakia. The eyes of cases 2, 3, and 4 had a history of trabeculectomy prior PMS implantation. The secondary open-angle glaucoma in the eyes of case 1 and case 4 was caused by cataract surgery, and in the eye of case 1, the intraocular lens was fixed in the sulcus due to rupture the posterior capsule. The eye of case 2 with primary angle-closure glaucoma had a history of cataract surgery, and the condition was recognized as an open-angle mechanism. The eye of case 3 underwent open bleb revision two months after PMS implantation. None of the eyes were treated with glaucoma medication at the time of needling.

**Figure 1:**
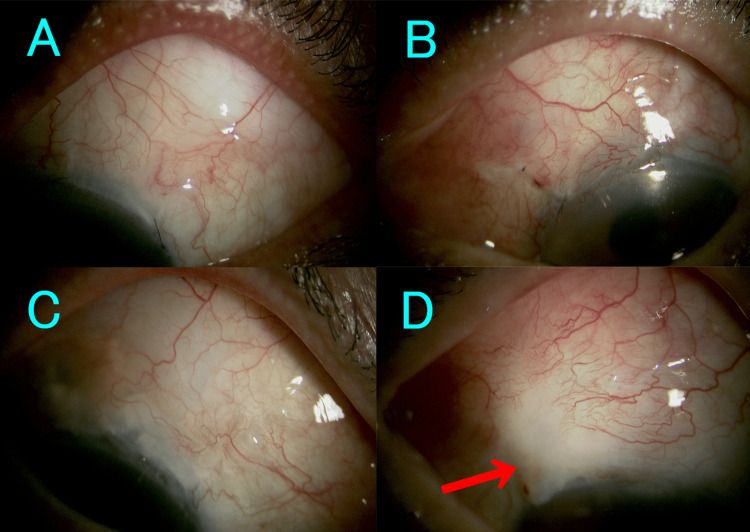
The bleb findings with filtration failure by slit lamp microscope before needling A represents the eyes of case 1, B the eye of case 2, C the eye of case 3, and D the eye of case 4. The eye of case 4 has partially avascular areas near the limbus (arrow).

Figure 1 shows the findings of slit-lamp microscope before needling. All eyes had a thick-walled vascular bleb, while there were partially avascular areas near the limbus in the eye of case 4.

Method

After applying 4% lidocaine ophthalmic solution (Xylocaine 4%) for topical anesthesia, the following procedures were performed in the operating room under surgical microscopy. First, a 1-mm side port was created in the corneal limbus, and an anterior chamber maintainer was inserted. Next, the position of the posterior end of the tube was estimated by measuring 8.5 mm from the tip of the tube. A bent 27-gauge needle was inserted into the conjunctiva from a position lateral to the target, and the needle tip was advanced near the posterior end of the tube, taking care not to touch the tube. Then, while maintaining continuous irrigation of the anterior chamber with a balanced salt solution (BSS) adjusting IOP to 20-36 mmHg by cataract surgery instrument, the fibrotic adhesions were lysing. During the procedure, the magnification of the surgical microscopy was adjusted to observe the tube in the anterior chamber, and the needle was moved while constantly paying attention to the tube’s behavior, especially movements that could pull it out of the anterior chamber. The procedure was continued for a few minutes, and then the needle was removed. The insertion site of the needle was not sutured. The anterior chamber maintainer was removed, and the side port was hydrated with BSS to confirm that there was no aqueous humor leakage (Video 1).

**Video 1:** Needling procedure with continuous infusion into the anterior chamber The video shows the findings of the eye of case 4

After needling, moxifloxacin and 0.1% betamethasone ophthalmic solutions were administered four times a day for one week, and then the frequency of administration was gradually reduced depending on the ocular findings. The patients were examined at day 1 and 1 week after needling, and thereafter every two to four weeks.

Results

In all cases, the formation of a filtering bleb immediately during the needling procedure, which is often seen with needling after trabeculectomy, was not observed. There was no complication during the needling procedure.

**Figure 2:**
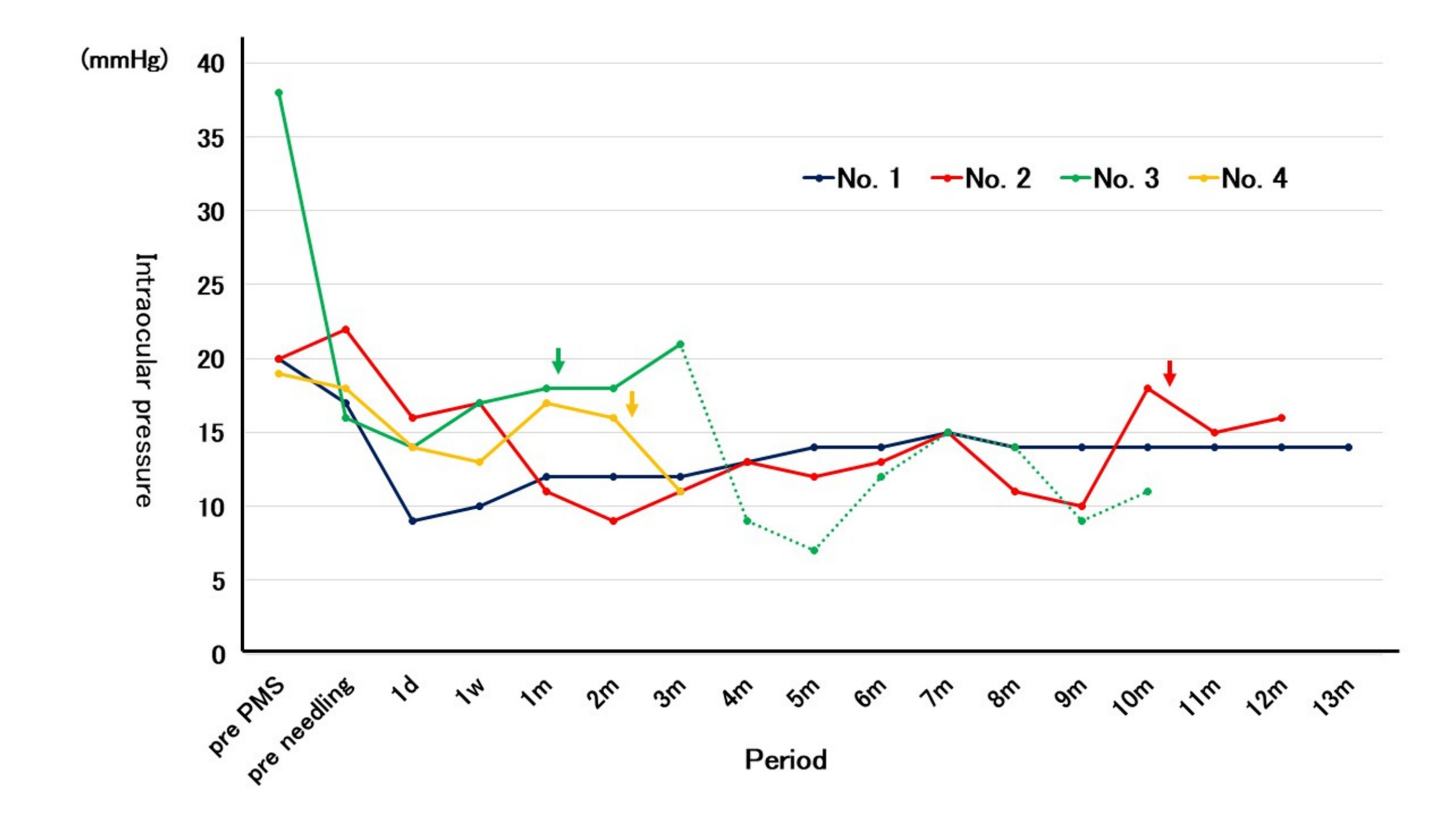
Intraocular pressure changes in each eye The arrow indicates repeated needling. The dotted line shows the change in the addition of intraocular pressure-lowering medication. PMS; PreserFlo MicroShunt implantation

The IOP changes in each eye are shown in Figure 2, and the individual clinical course is shown in Table 2.

**Table 2:** Individual Clinical Course BCVA; best-corrected visual acuity, ECD; endothelial cell density, IOP; intraocular pressure

No.	Follow-up period (month)	BCVA at the last visit (logMAR)	ECD at the last visit (cell/mm^2^)	Additional procedure (s) (time point of additional procedure)
1	13	0	2,488	-
2	12	0.1	1,327	Repeat needling (10 months)
3	10	0	2,050	Repeat needling (1 month), addition of IOP-lowering medication (3 months)
4	3	0	2,404	Repeat needling (2 months)

**Figure 3:**
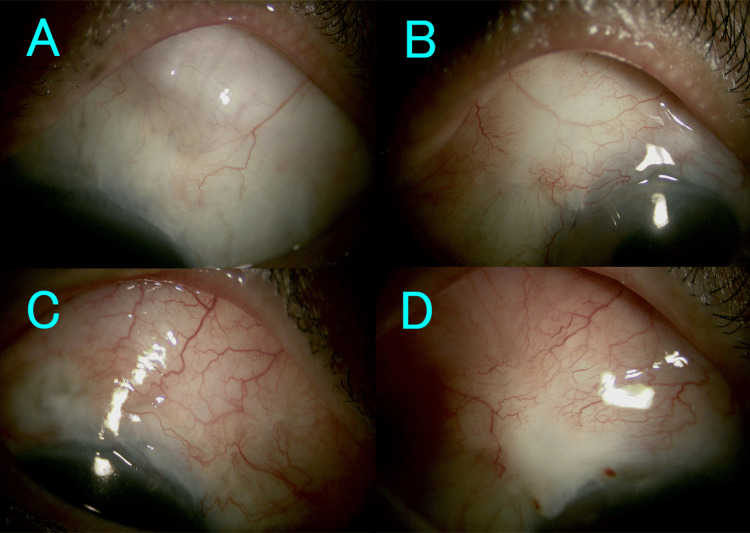
Slit-lamp microscopy findings at the last visit after needling A represents the eye of case 1, B the eye of case 2, C the eye of case 3, and D the eye of case 4. Bleb formation is maintained; however, only the eye of case 3 shows significant hyperemia due to the intraocular pressure-lowering medication.

The eye of case 2 required a repeat needling 10 months after the initial procedure. Case 3's eye required a repeat needling one month after the initial procedure, as well as the addition of IOP-lowering medication three months after the procedure. Case 4's eye required a repeat needling at two months. Except for the eye of case 3, the addition of IOP-lowering medication was not necessary during follow-up. No eyes required additional glaucoma surgery. Slit-lamp microscopy findings at the last visit after needling are shown in Figure 3. No ocular infection occurred throughout the entire clinical course.

## Discussion

This study evaluated the efficacy and safety of needling with anterior chamber irrigation for treating filtration failure after PMS implantation in a small number of cases. While a large reduction in IOP was not achieved, many cases maintained lower IOP than before surgery, and some did not require additional eye drops. These results suggest that combining anterior chamber irrigation with conventional needling techniques may have certain clinical significance.

Many clinicians must believe that post-PMS implantation needling yields less filtration effect than post-trabeculectomy needling. In post-trabeculectomy needling, filtering bleb forms immediately after lysis of adhesion. In contrast, post-PMS implantation needling rarely shows filtering bleb formation, even when adhesions are lysed. This is because the PMS outflow pathway for aqueous humor is pinpoint, limited solely to the 70-μm inner-diameter opening at the tube's distal end [1], resulting in restricted filtration volume. Given this structural characteristic, it is reasonable to conclude that needling after PMS implantation using the same method as after trabeculectomy is unlikely to be effective in cases of filtration failure.

There are reports that combining infusion with needling can visualize the filtering effect intraoperatively, making it easier to determine the timing for ending the procedure in cases of filtration failure after trabeculectomy [8,9]. I considered applying this method to post-PMS implant needling to be reasonable and attempted it, achieving success in some cases. Unfortunately, however, I was unable to visualize sufficient filtration. This suggests that the structural limitations of the PMS still exert an influence, even with anterior chamber perfusion. Moving forward, it will be necessary to explore more effective methods, such as temporarily increasing the perfusion pressure.

European physicians with extensive experience with PMS implantation agree that open revision is preferred over bleb needling when target IOP is not maintained or when filtration failure occurs postoperatively [6,7]. However, since reoperation is highly invasive, it should be avoided whenever possible for the surgeon and the patient. In fact, a systematic review reported no difference in needling rates after PMS implantation and trabeculectomy [2]. The only prospective, multicenter randomized controlled trial reported that needling was required in 98 (25%) of 395 eyes in the PMS implantation group and in 12 (9%) of 132 eyes in the trabeculectomy group, indicating significantly more needling in the PMS implantation group [10]. These findings support the fact that, though not recommended, needling is commonly attempted postoperatively after PMS implantation. Clinicians desire effective needling techniques, yet previous reports lack descriptions of specific needling methods. Furthermore, no reports detail the postoperative course following needling, such as the proportion of eyes requiring reoperation surgery among those treated with needling. This report focuses on a novel technique with the potential to improve needling outcomes after PMS implantation.

The techniques described in this report represent an improvement over the conventional needling technique used after trabeculectomy. Needling with continuous perfusion into the anterior chamber requires a surgical microscope. However, the thickened bleb wall after PMS implantation prevents visualization of the tube with a surgical microscope. The tube length is 8.5 mm; therefore, the procedure was performed while estimating the distal end position of the tube using calipers. This method helps avoid randomly moving the needle during the procedure when the posterior end of the tube cannot be identified. Since the tube may be curved beneath the tissue, it may be beneficial to confirm its position using anterior segment optical coherence tomography beforehand [11].

The subconjunctival injection of a local anesthetic can alter the original elevated morphology of the filtration bleb, which can make it difficult to determine the direction and extent of adhesion release during needling. Therefore, topical anesthesia was used in these eyes. While pain control was achieved in all cases, subconjunctival anesthesia may be necessary if severe pain prevents continuation of the procedure. Thorough preoperative orientation is essential to ensure this, and the amount of anesthetic injected should be kept to a minimum.

There has been no unified consensus on the site of needle insertion or the direction of advancement. This report describes a procedure in which the needle is inserted just lateral to the filtration bleb. Adhesions are peeled off under magnification with a surgical microscope, which allows visualization of the subconjunctival portion of the needle tip and the tube tip within the anterior chamber. This approach prevents complications in which the needle tip becomes snagged on the tube and is pulled out [12]. The goal of PMS implantation is to direct the filtered aqueous humor toward the fornix. Therefore, it is necessary to verify the optimal needle insertion site and direction for the adhesion lysis procedure. Infusion into the anterior chamber is likely to be effective in this verification process as well.

This method has several limitations. First, although not observed in this series, there are risks of infection and corneal endothelial damage associated with intraocular manipulation. Additionally, since anterior chamber infusion is required, the procedure must be performed in an operating room. Consumption of medical resources is also significant, including equipment for continuous perfusion, disposable supplies, and sterilization/disinfection of instruments. Conversely, slit-lamp needling is simple and can be performed in facilities without operating rooms. In Japan, insurance claims cannot be made for combining needling with continuous infusion; therefore, the additional cost falls on the medical facility. The greatest limitation of this report is that it is a case series with a small number of eyes and no comparison group. Future verification with a larger sample size and long-term evaluation are necessary.

## Conclusions

This report on needling under anterior chamber infusion for postoperative filtration failure following PMS implantation frames the findings as preliminary feasibility data showing potential safety and utility of needling outcomes. In this small study, all patients avoided additional surgery, suggesting the technique's potential clinical utility. However, challenges remain, including eyes in which adequate filtration was not achieved, risks specific to intraocular surgery, and the strain on healthcare resources.

At present, needling with continuous infusion into the anterior chamber cannot be considered the primary procedure for post-PMS implantation filtration failure. However, performing continuous infusion using the anterior chamber maintainer while elevating IOP and lysing the fibrotic adhesions is a logical approach to increasing the filtration volume of a device with a limited flow and a 70-μm lumen diameter. It may be a useful option when open bleb revision must be avoided. Moving forward, additional comparative studies to conventional needling, as well as evaluation of long-term outcomes, are required to establish its efficacy and safety.
